# Recent infections among individuals with a new HIV diagnosis in Rwanda, 2018–2020

**DOI:** 10.1371/journal.pone.0259708

**Published:** 2021-11-17

**Authors:** Gallican N. Rwibasira, Samuel S. Malamba, Gentille Musengimana, Richard C. M. Nkunda, Jared Omolo, Eric Remera, Vedaste Masengesho, Valens Mbonitegeka, Tafadzwa Dzinamarira, Eugenie Kayirangwa, Placidie Mugwaneza

**Affiliations:** 1 Mailman School of Public Health, International Centre for AIDS care and Treatment program (ICAP) at Columbia University, Kigali, Rwanda; 2 Division of Global HIV/AIDS & TB, Centers for Global Health, US Centers for Disease Control and Prevention, City of Kigali, Rwanda; 3 Department of HIV, AIDS, Diseases prevention and Control, Division of HIV, STI, Viral Hepatitis and Other Viral Diseases Control, Ministry of Health, Rwanda Biomedical Centre (RBC), Kigali, Rwanda; Hebei provincial center for disease control and prevention, CHINA

## Abstract

**Background:**

Despite Rwanda’s progress toward HIV epidemic control, 16.2% of HIV-positive individuals are unaware of their HIV positive status. Tailoring the public health strategy could help reach these individuals with new HIV infection and achieve epidemic control. Recency testing is primarily for surveillance, monitoring, and evaluation but it’s not for diagnostic purposes. However, it’s important to know what proportion of the newly diagnosed are recent infections so that HIV prevention can be tailored to the profile of people who are recently infected. We therefore used available national data to characterize individuals with recent HIV infection in Rwanda to inform the epidemic response.

**Methods:**

We included all national-level data for recency testing reported from October 2018 to June 2020. Eligible participants were adults (aged ≥15 years) who had a new HIV diagnosis, who self-reported being antiretroviral therapy (ART) naïve, and who had consented to recency testing. Numbers and proportions of recent HIV infections were estimated, and precision around these estimates was calculated with 95% confidence intervals (CI). Logistic regression was used to assess factors associated with being recently (within 12 months) infected with HIV.

**Results:**

Of 7,785 eligible individuals with a new HIV-positive diagnosis, 475 (6.1%) met the criteria for RITA recent infection. The proportion of RITA recent infections among individuals with newly identified HIV was high among those aged 15–24 years (9.6%) and in men aged ≥65 years (10.3%) compared to other age groups; and were higher among women (6.7%) than men (5.1%). Of all recent cases, 68.8% were women, and 72.2% were aged 15–34 years. The Northern province had the fewest individuals with newly diagnosed HIV but had the highest proportion of recent infections (10.0%) compared to other provinces. Recent infections decreased by 19.6% per unit change in time (measured in months). Patients aged ≥25 years were less likely to have recent infection than those aged 15–24 years with those aged 35–49 years being the least likely to have recent infection compared to those aged 15–24 years (adjusted odds ratio [aOR], 0.415 [95% CI: 0.316–0.544]).

**Conclusion:**

Public health surveillance targeting the areas and the identified groups with high risk of recent infection could help improve outcomes.

## Introduction

Considerable progress has been made to contain the HIV epidemic globally. However, data reported from the Joint United Nations Programme on HIV/AIDS (UNAIDS) show the need to keep the momentum to achieve sustainable epidemic control by 2030 [1, 2]. New HIV infections have decreased globally, due in part to scale-up of antiretroviral therapy (ART) coverage, early ART initiation and retention of individuals with new HIV diagnoses, and viral load (VL) suppression, which help decrease transmission rates [3, 4].

Despite this progress, in 2017, an estimated 14.5 million people had undiagnosed HIV infection [5]. To address this gap, new strategies, including active case finding and recency testing, were integrated in many country programs with limited HIV-testing resources to identify high-risk individuals with undiagnosed HIV infections and help routinely detect and interrupt HIV transmission networks to achieve epidemic control. In 2018, Rwanda adopted recency testing to monitor what percentage of the newly diagnosed HIV positives was recent. This was intended to inform HIV prevention and HIV testing to target people who are likely to acquire and transmit HIV [6]. Recency testing with limiting-antigen avidity assays is used in some African countries to estimate HIV incidence and has been proven to be cost-effective in estimating new infections to inform the public health response [7]. New HIV infections in Rwanda previously have been estimated using mathematical modeling from the Estimation and Projection Package-Spectrum and cohort-based surveys [8]. Recency testing could be another method to estimate HIV incidence in other countries, including Rwanda. It may also be useful for index testing and finding partners who may also be recently infected but it is not intended for the diagnosis of HIV infection.

Despite Rwanda’s Progress toward HIV epidemic control, 16.2% of HIV-positive individuals are unaware of their HIV positive status [9]. Tailoring the public health strategy could help reach these individuals with new infection and achieve epidemic control. In 2018, the Rwanda Ministry of Health and its partners launched a 5-year strategic plan to address new HIV infections to help the country achieve and sustain HIV epidemic control [10]. In October 2018, Rwanda introduced laboratory methods used to detect and confirm recent infections among individuals with a new HIV diagnosis within the last 12 months [7]. These new approaches have helped the government of Rwanda to increase awareness of HIV status among PLHIV, to enhance timely linkage to care and treatment, and to stop transmission under the case-based surveillance program. This approach has helped map new HIV infections and characterize individuals at high risk of infection to develop tailored prevention measures to stop HIV transmission. In this study, we used data from the Recency Web application to evaluate the progress made in identifying recent HIV infections and identify risk factors associated with recent HIV infection.

## Methods

### Study design

Our retrospective cross-sectional study used recency testing data collected through the Rwanda National Health Information System (October 2018–June 2020). Recency data are collected using an electronic system called the Recency Application (known as the Recency App) and Laboratory Information Management System (Web LIMS/LabWare). The system also collects data on patient identification, quality control, demographic information including age and Sex, and health facility information. Collected information is uploaded into the Recency App and Web LIMS/LabWare, and a countrywide dataset was exported for our analysis.

### Data collection

Clients with HIV-positive results from voluntary counselling and testing services, out-patient departments, antenatal care, voluntary medical male circumcision program, internal patient departments, and prevention of mother-to-child HIV transmission program are screened for eligibility for recency testing. Our analysis included patients aged ≥15 years with a new HIV diagnosis made during the study period (October 2018–June 2020) and, self-reported that they had never initiated ART. Participants provided verbal informed consent (materials were written in English and were translated into Kinyarwanda, the local language). Clients who did not provide informed consent received HIV care and treatment, per national guidelines.

### Laboratory testing

**Fig 1:**
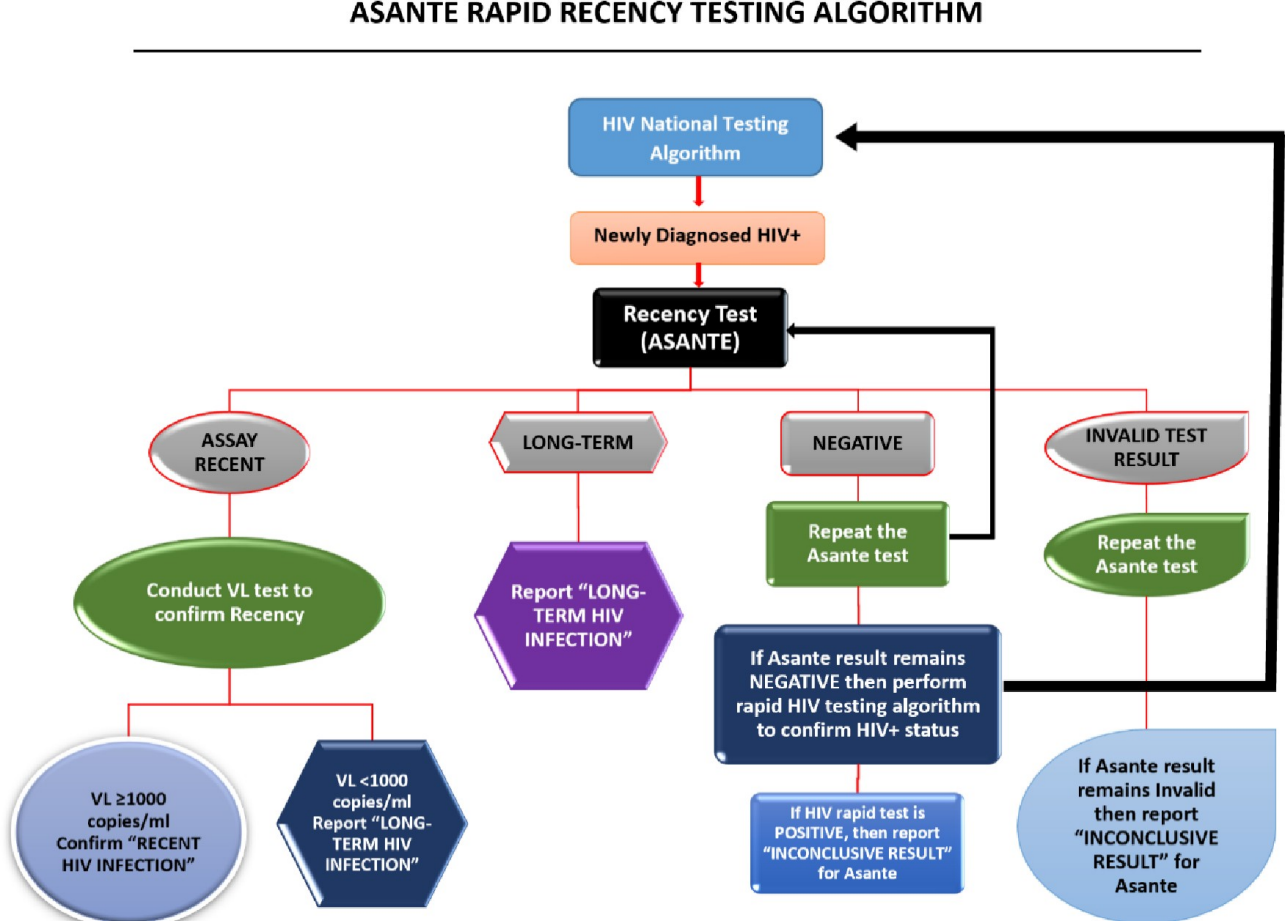
The Asante Rapid Test and Recent Infection Testing Algorithm (RITA).

The Asante rapid HIV-1 recency test assay based on limited antigen and antibody avidity was used to perform recency testing on clients with newly diagnosed HIV-1 who were aged ≥15 years and who had received counselling and had given informed consent before initiating ART. Whole-blood samples were collected from eligible clients at primary health care facilities. Rapid recency tests were performed, per the recency testing standard operating procedure, by trained laboratory staff at primary health care facilities or at VL testing hubs (e.g., the Rwanda National Reference Laboratory) located across the country. According to the Asante assay, the duration of infection flagged as recent is ≤12 months. The False Recency Rate (FRR) was estimated to be about 1% excluding treated patients and elite controllers [11]. Additional recent validation of the assay to generate more data in specimens from individuals of known duration of infection have shown a very high specificity but lower sensitivity in Uganda [12] and very high for both in Vietnam [13]. Long-term infection results were immediately returned to the participants, and all samples identified as recent infection via Asante underwent additional testing for VL, per the Recent Infection Testing Algorithm (RITA) in Rwanda (Fig 1). Pre- and post-test counseling on what ‘recent infection’ means and implication of recency results for their own health as well as health of sexual partners was provided to all participants classified as recent.

VL testing was conducted using Roche COBAS AmpliPrep/COBAS TaqMan HIV-1 for plasma samples. Samples with VL ≥1000 copies/mL were confirmed as recent infection, and those with <1000 copies/mL were reclassified as long-term infections. RITA results were returned to the health facility within 14 days. Results confirming recent infection and VL were returned to the client.

All laboratory test results, including quality control and individual sample testing, were recorded in the laboratory records and uploaded into the Recency App and LIS, respectively. The National Reference Laboratory periodically reviews completed data from testing sites and reporting to the program for surveillance and continuous quality improvement at health facilities.

### Ethical consideration

Recency testing was approved under the Active Case-Based Surveillance for HIV in Rwanda protocol. The program was reviewed and approved by the Rwanda National Ethics Committee. It was also reviewed in accordance with CDC human research protection procedures and was determined to be research, but CDC investigators did not interact with human subjects or have access to identifiable data or specimens for research purposes.

All eligible patients provided verbal informed consent before enrolling in the study. A waiver of parental consent and assent was received from the Rwanda National Ethic Committee for participants aged 15–17 years who provided verbal consent before enrollment into the program.

### Statistical analysis

We determined the number of recent HIV infections among clients with newly diagnosed HIV, estimated and tested for the difference in proportions using chi-square test for bivariate comparison, and calculated 95% confidence intervals (CI). Logistic regression was used to identify risk factors associated with recent HIV infection. Factors with p≤0.1 on univariate logistic regression were included in a full model using stepwise regression in which final explanatory variables were chosen automatically: in each step, a variable was considered for addition to or subtraction from this set of explanatory variables based on the prespecified criterion of including factors that were associated with recent HIV infections at p<0.05 in the final multivariable logistic regression model.

All proportion estimates were weighted to account for non-acceptance to recency testing and reporting gaps among individuals with newly diagnosed HIV at the time of using the Recency App. A weight was calculated by taking the reciprocal of the proportion of all individuals with newly diagnosed HIV reported through the Recency App during the reporting period.

Time trends were analyzed using logistic regression with recent infection as a dependent variable and time in month as an independent variable. Multivariable logistic regression with recent infection as a dependent variable was fitted to measure the association between HIV recency and sex, key age groups, and provinces. Independent categorical variables included a missing value category to minimize list-wise deletion of observation in the models. Data were managed and analyzed using STATA, version 15.0 (College Station, TX).

## Results

**Fig 2:**
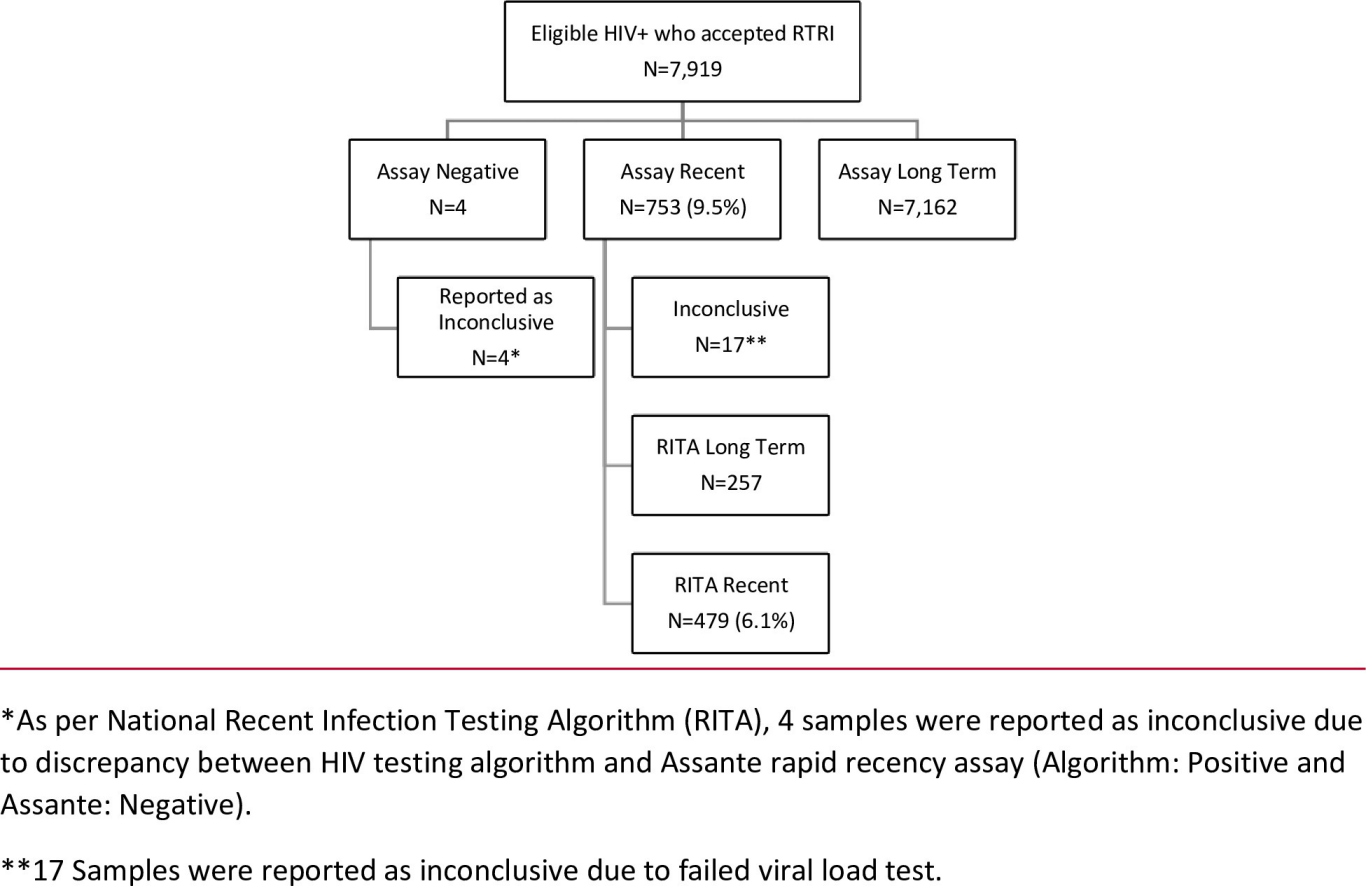
Flow diagram of final HIV recency testing outcome by assay outcome in Rwanda (2018–2020).

Between October 2018 and June 2020, a total of 1,845,061 samples were tested for HIV in the sites that were offering recency testing at the time, of which 1,834,360 (99.4%) tested HIV negative, and 10,701 (0.6%) tested HIV positive. Of the positive samples, approximately a quarter (2,782) were never tested for recency due to two main reasons, namely having no consent for recency testing, and finding evidence during the recency testing counseling sessions of prior exposure to antiretroviral drugs. A total of 7,919 samples (74%) were sent for recency assay testing, of which 753 tested assay recent, giving a proportion of assay recent infections of 9.5% (753/7919) but when recent infection was defined as assay recent together with a viral load (VL) ≥1,000 copies/mL (excluding the negative and, inconclusive sample results), only 6.1% (479/7898) of samples were classified as RITA recent infections (Fig 2).

The absolute number of recent infections among men and women were highest among women aged 15–34 years (259) and among men aged 25–49 years (108). The proportion of recent infections among newly identified positives was higher among women (6.7%) than men (5.1%) and highest in both men (10.0%) and women (9.5%) aged 15–24 years (Table 1). Though the number of samples tested was small, a high proportion (10.3%) of the newly identified HIV positive samples from men aged ≥65 years tested recent. Of all recent cases, 68.8% (327/475) were women, and 72.2% (343/475) were aged 15–34 years.

**Table 1:** HIV RITA recency testing outcome by age and sex in Rwanda (2018–2020)*.

	Women			Men			Combined		
Age, years	Total	Long Term (%)	Recent (%)	Total	Long Term (%)	Recent (%)	Total	Long Term (%)	Recent (%)
15–24	1,338	1,211 (90.5)	127 (9.5)	259	233 (90.0)	26 (10.0)	1,597	1,444 (90.4)	153 (9.6)
25–34	1,999	1,867 (93.4)	132 (6.6)	1,110	1,052 (94.8)	58 (5.2)	3,109	2,919 (93.9)	190 (6.1)
35–49	1,203	1,154 (95.9)	49 (4.1)	1,206	1,156 (95.8)	50 (4.2)	2,409	2,310 (95.9)	99 (4.1)
50–64	297	279 (93.9)	18 (6.1)	272	264 (97.1)	8 (2.9)	569	543 (95.4)	26 (4.6)
65+	43	42 (97.7)	1 (2.3)	58	52 (89.7)	6 (10.3)	101	94 (93.1)	7 (6.9)
Total	4,880	4,553 (93.3)	327 (6.7)	2,905	2,757 (94.9)	148 (5.1)	7,785	7,310 (93.9)	475 (6.1)

*Age or sex were not recorded on 113 samples, (long-term, 105; recent, 8), 4 samples tested negative and 17 samples did not have a conclusive recency test result. They were therefore excluded from analysis and results presented in Table 1.

**Fig 3:**
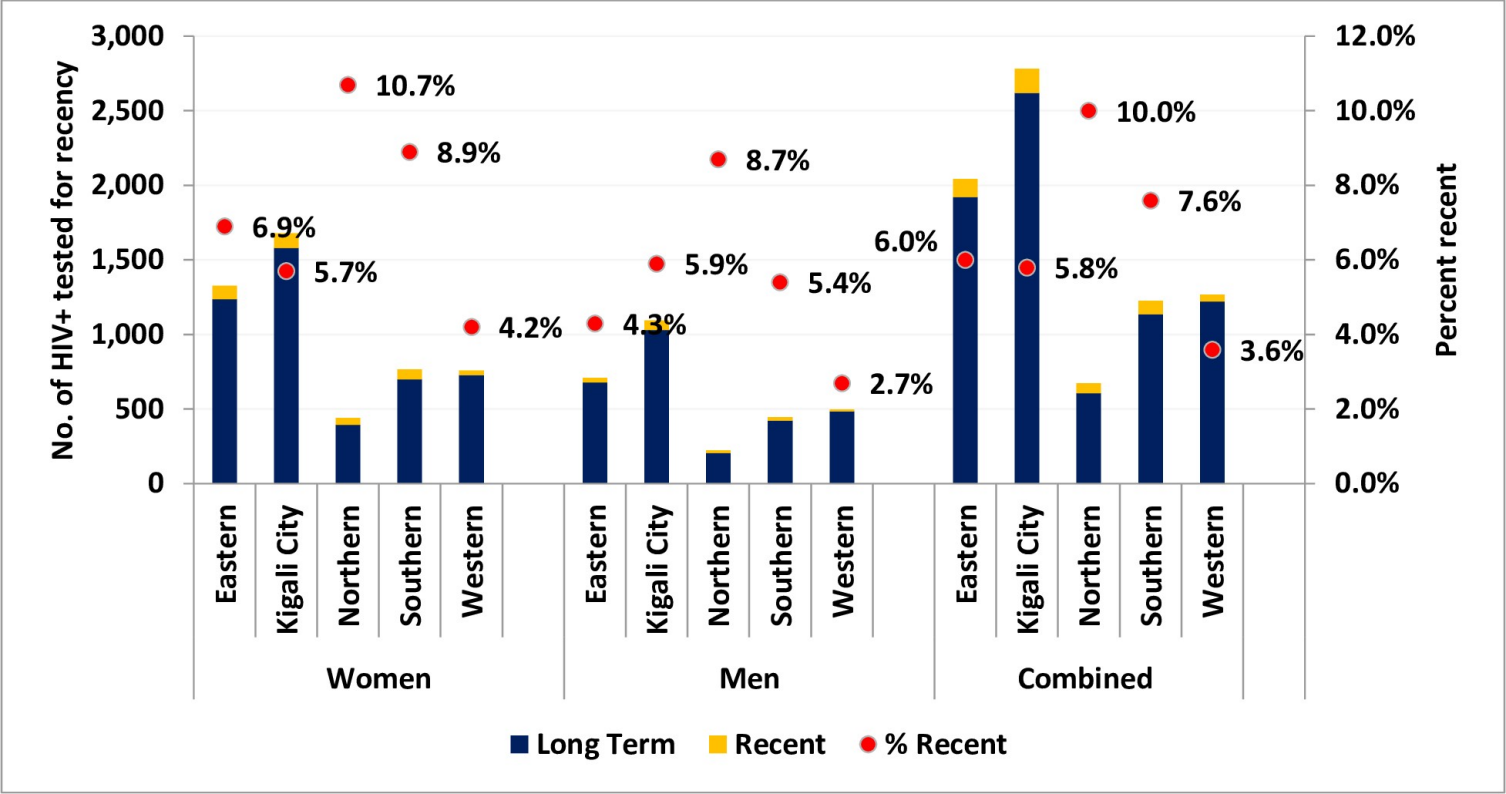
Newly diagnosed and recent HIV infections by province in Rwanda (2018–2020).

The pattern of newly diagnosed and recent HIV infections by province was similar between men and women, though the absolute number of recent infections was higher among women than men. For both men and women, the proportion of recent infections was highest in the Northern province, which had the lowest number of newly diagnosed HIV infections. The City of Kigali considered the fifth province and the capital city of Rwanda) had the highest numbers of newly diagnosed HIV infections (Fig 3).

**Fig 4:**
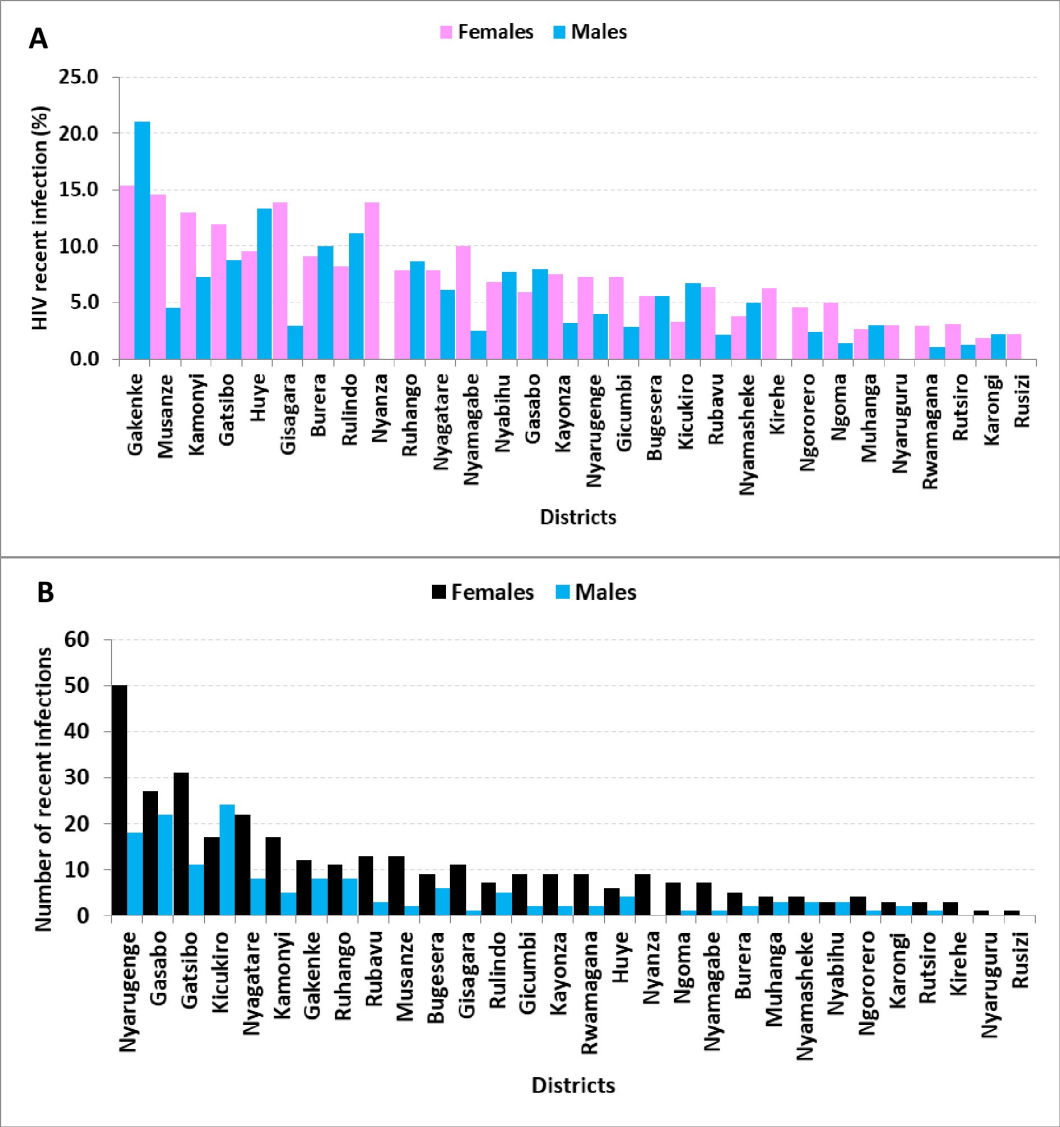
A. Indicating the final recent HIV infection proportions and B. Indicating the absolute number of recent HIV infections by district and sex in Rwanda (2018–2020).

Five of the top ten districts with the highest proportion of recent infections in newly diagnosed HIV infections were from the Northern Province (Fig 4A), and the three districts making up the City of Kigali had the highest number of absolute recent infections (Fig 4B).

**Fig 5:**
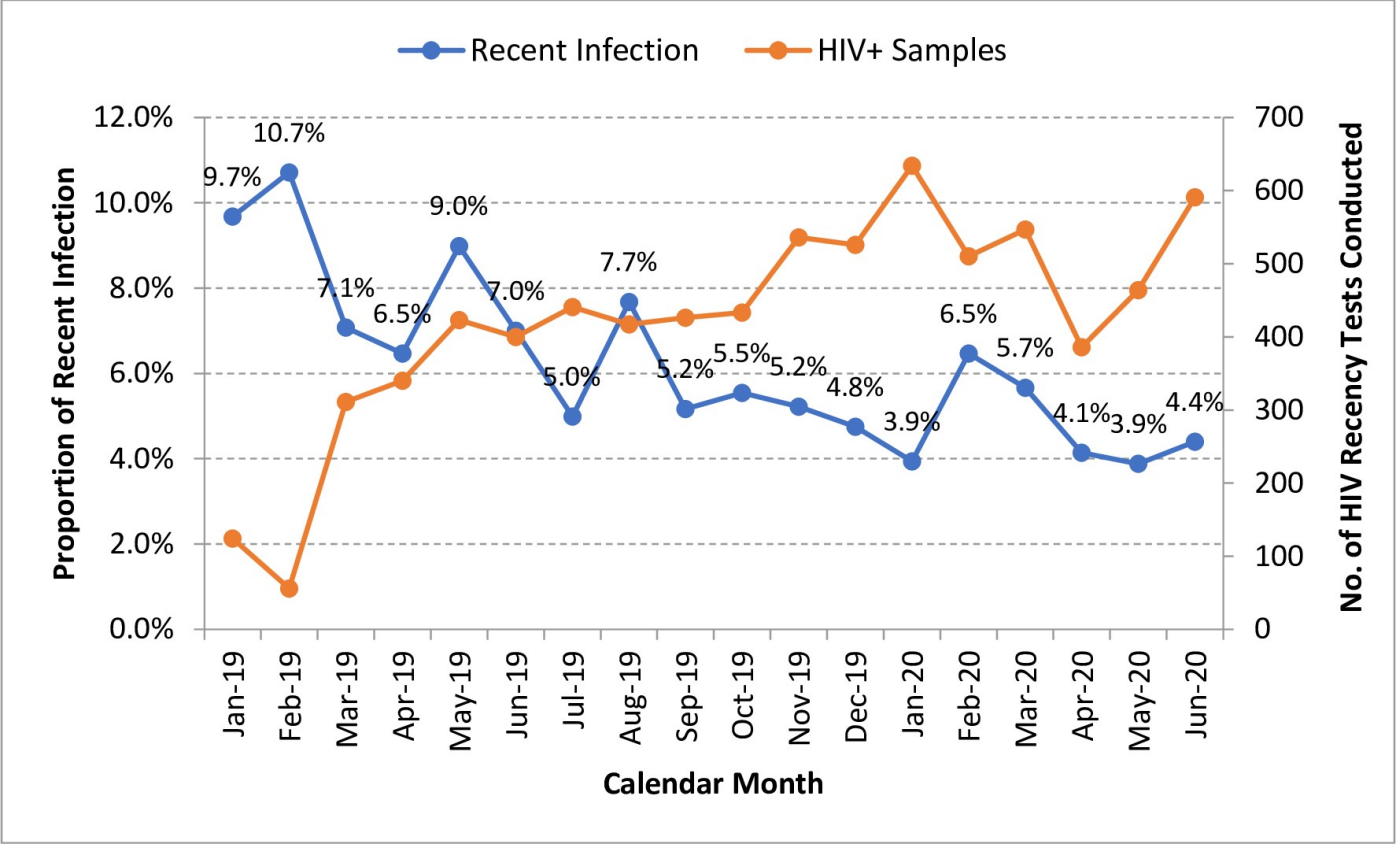
Proportion of recent infections per month among individuals with newly identified HIV infection in Rwanda (2018–2020).

The proportion of recent infections among individuals with newly identified HIV infection in Rwanda decreased, but the number of HIV-positive samples tested for recent infections increased over time (Fig 5).

After adjusting for differences in sex, age, and province where samples with newly identified HIV infection were collected, we observed a significant decrease in individuals with recent HIV infection (5.2% per month; adjusted odds ratio [aOR], 0.948; Table 2).

**Table 2:** Univariable and multivariable logistic regression analysis of recent infection with sex, age, and province in Rwanda (2018–2020).

Variable	N = 7,785	No. RITA recent (%)	Unadjusted Odds Ratio	95% CI	p-value	Adjusted Odds Ratio	95% CI	p-value
**Time in Quarters**	7,785	475 (6.1%)	0.830	0.785–0.878	<0.001	0.804	0.757–0.853	<0.001
**Sex**								
Female	4,880	327 (6.7%)	1.000			1.000		
Male	2,905	148 (5.1%)	0.747	0.612–0.913	0.004	0.883	0.716–1.089	0.243
**Age, years**								
15–24	1,597	153 (9.6%)	1.000			1.000		
25–34	3,109	190 (6.1%)	0.614	0.492–0.767	<0.001	0.625	0.497–0.784	<0.001
35–49	2,409	99 (4.1%)	0.404	0.311–0.525	<0.001	0.415	0.316–0.544	<0.001
50–64	569	26 (4.6%)	0.452	0.295–0.693	<0.001	0.449	0.290–0.694	<0.001
≥65	101	7 (6.9%)	0.703	0.320–1.542	0.379	0.752	0.339–1.666	0.482
**Provinces**								
Eastern	2,014	120 (6.0%)	1.000			1.000		
City of Kigali	2,726	157 (5.8%)	0.965	0.756–1.232	0.773	0.761	0.587–0.985	0.038
Northern	650	65 (10.0%)	1.754	1.279–2.405	<0.001	1.560	1.133–2.147	0.006
Southern	1,175	89 (7.6%)	1.293	0.974–1.719	0.076	1.189	0.891–1.587	0.240
Western	1,220	44 (3.6%)	0.591	0.415–0.841	0.003	0.527	0.369–0.752	<0.001

* Samples with inconclusive recency test result were excluded from this analysis.

Individuals with newly identified HIV aged 15–24 years were more likely to have recent infection than those aged 25–34 years (aOR = 0.625 [95% CI: 0.497–0.784]), 35–49 years (aOR = 0.415 [0.316–0.544]) and 50–64 years (aOR = 0.449 [95% CI: 0.290–0.694). Individuals with newly identified HIV from the Eastern Province were more likely to have recent infection than those from the Western Province (aOR, 0.527 [95% CI: 0.369–0.752]) or from the City of Kigali (aOR, 0.761 [95% CI: 0.587–0.985]). HIV-positive individuals from the Northern Province were more likely to have a recent infection than those from the Eastern Province (aOR, 1.560 [95% CI: 1.133–2.147]), even though the Northern Province had the fewest number of individuals with newly identified HIV infections (Table 2).

## Discussion

Our study is the first to analyze recency data 2 years after Rwanda launched recency testing to map and characterize new infections and inform public health strategies. We found that 6.1% of PLHIV with a new diagnosis had acquired this infection within the last 12 months of sample collection. This finding is similar to the recency rate reported in a feasibility study done in Kenya (8.6%) but is lower than rates reported in European studies that mainly focused on high-risk groups [14]. The limiting antigen avidity assay methods have been used to measure HIV incidence [7] however, the Asante rapid recency assay testing, though based on the same principle as the limiting antigen (LAg) avidity assay, was used for surveillance and monitoring trends of active transmission of HIV in the population and to inform epidemic response in Rwanda.

We found that the biggest number of recent infections were localized in the City of Kigali, compared to other provinces. The City of Kigali has more female sex workers and men who have sex with men than other provinces in Rwanda [15–17], and these key populations have higher HIV infection rates and more recent infections than in the general population. The City of Kigali also has the highest HIV prevalence (3.7%) in Rwanda compared to other provinces (Southern, 2.3%; Western, 2.8%; Northern, 1.8%; and Eastern, 2.5%) [9] and thus has high rates of transmission [8, 9]. The City of Kigali also may have had more recent infections because, as the pilot site before national implementation, health facilities in the City of Kigali received more mentorship to implement recency testing than other sites. The City of Kigali has a higher HIV-testing volume than other provinces because residents are more likely to get tested for HIV than Rwandans in other provinces due to more access to HIV-testing locations and education about HIV prevention [9].

Our finding that women represent 70% of the total HIV recent infections compared to men supports other reported data [18]. This finding might be explained by gender discrepancies in seeking healthcare services, by women’s higher HIV acquisition risk, or imbalances in power in negotiating for safe sex. However, the odds of testing recent in females, was not statistically significant from that in males.

Our findings show that recent HIV infections were more frequent in women aged 15–34 years and among men aged 25–49 years. The odds of testing recent was lower among those aged ≥25 years compared to those aged 15–24 years. Similar findings have been reported in other studies where young women have the most recent infections [18]. In the 2018 Rwanda Population-Based HIV Impact Assessment (RPHIA) survey, young women aged 20–24 years had the highest HIV prevalence, which was 3 times higher than men of the same age; the HIV prevalence was higher in men aged ≥50 years than in women of the same age group [9]. The results observed in this study and method of characterizing the recent HIV infection are in line with what was found elsewhere [6, 14, 18, 19].

Unlike the RPHIA results [9], our findings suggest that rural areas have a higher proportion of absolute number of recent infections, despite having lower testing rates, than urban areas. This observation may be related to an increase in recent HIV infections in other provinces compared to the City of Kigali, which aligns with RPHIA findings [9]. Another reason might be that rural areas (2.1%) in Rwanda have lower HIV prevalence than urban areas (4.1%), but urban areas have higher ART coverage and awareness of HIV-positive status than rural areas (indicated by the average number of people who know their HIV status and are receiving ART: urban, 80.2%; rural, 77.4%) [9]. The decrease in recent HIV infections to 5.2% after adjusting for age, sex, and province might have been due to scale-up of recency testing in rural health facilities with low HIV prevalence.

Recency testing is one of the strategies used in active case finding to detect new HIV transmission. In Rwanda, national guidelines recommend that every person with newly diagnosed HIV should be offered index testing and recency testing in addition to the standard of care. Studies conducted elsewhere revealed that new strategies such as active case finding can help individuals with a recent HIV-positive diagnosis to disclose their status to their partners and can increase the yields of the HIV-positive individuals who did not know their status and help them initiate ART [5, 20]. Additionally, contacts with HIV-negative results are counselled and linked to HIV prevention measures to maintain their HIV-negative serostatus [21].

Our study has several limitations. First, HIV recency testing is offered to individuals with a new HIV diagnosis; because only consenting individuals are tested for recency, our study sample does not include all individuals with a new HIV diagnosis. Second, Rwanda does not have a national unique identification system for HIV-positive individuals, so an individual with recent infection may be tested at different facilities and recorded more than once. However, because recency testing was offered only to individuals with a new HIV diagnosis, the number of repeat testers was small. Thirdly, some recent infections may have to be reclassified as long term if testing shows VL suppression, possibly because the client did not disclose their history of prior ART or is an elite VL controller. Lastly, ARV naivety was based on self-report as opposed to ART metabolite testing and this could have resulted in misclassification of recency by the assay.

We found that across all age groups, women had more absolute recent HIV infections than men. Individuals aged 15–34 years and men ≥65 years had a high prevalence of recent HIV infection. The City of Kigali had a higher rate of recent infections than other provinces. These findings warrant increased prevention interventions (e.g., HIV testing, condom distribution and education, and behavior change communication) targeting these individuals and introduction of pre-exposure prophylaxis among HIV-negative sexual partners of individuals with a new HIV diagnosis. Findings from this study showed that once integrated in routine HIV-testing services, recency testing can help map, characterize, and manage individuals with newly diagnosed HIV. Adopting recency testing and other HIV-testing initiatives in epidemic surveillance could help inform targeted prevention measures.
